# A pilot, short-term dietary manipulation of branched chain amino acids has modest influence on fasting levels of branched chain amino acids

**DOI:** 10.3402/fnr.v60.28592

**Published:** 2016-01-14

**Authors:** Nicole Landa Cavallaro, Jamie Garry, Xu Shi, Robert E. Gerszten, Ellen J. Anderson, Geoffrey A. Walford

**Affiliations:** 1 Center for Human Genetic Research, Massachusetts General Hospital, Boston, MA, USA; 2 Metabolism & Nutrition Research, Clinical Research Center, Massachusetts General Hospital, Boston, MA, USA; 3 Harvard Catalyst Clinical Translational Science Center, Harvard Medical School, Boston, MA, USA; 4 Cardiovascular Research Center, Massachusetts General Hospital, Boston, MA, USA; 5 Cardiology Division, Massachusetts General Hospital, Boston, MA, USA; 6 Harvard Medical School, Boston, MA, USA; 7 Diabetes Research Center, Diabetes Unit, Department of Medicine, Massachusetts General Hospital, Boston, MA, USA

**Keywords:** branched chain amino acids, dietary intervention, nutritional modulation, cross-over clinical trial, metabolism, insulin sensitivity

## Abstract

**Background:**

Elevated fasting levels of branched chain amino acids (BCAAs: valine, isoleucine, leucine) in venous blood are associated with a variety of metabolic impairments, including increased risk of type 2 diabetes (T2D). Fasting BCAA levels are influenced by non-dietary factors. However, it is unknown whether fasting BCAAs can be altered through manipulation of dietary intake alone.

**Objective:**

To test whether a specific dietary intervention, using differences in BCAA intake, alters fasting BCAA levels independent of other factors.

**Design:**

Five healthy male volunteers underwent 4 days of a low and 4 days of a high BCAA content dietary intervention (ClinicalTrials.gov [NCT02110602]). All food and supplements were provided. Fasting BCAAs were measured from venous blood samples by mass spectrometry at baseline and after each intervention.

**Results:**

Diets were isocaloric; contained equal percentages of calories from carbohydrate, fats, and protein; and differed from each other in BCAA content (1.5±0.1 vs. 14.0±0.6 g for valine; 4.5±0.9 g vs. 13.8±0.5 g for isoleucine; 2.1±0.2 g vs. 27.1±1.0 g for leucine; *p<*0.0001 for all). Fasting valine was significantly lower (*p=*0.02) and fasting isoleucine and leucine were numerically lower following the low BCAA content vs. the high BCAA content diet levels. The inter-individual response to the dietary interventions was variable and not explained by adherence.

**Conclusion:**

Short-term dietary manipulation of BCAA intake led to modest changes in fasting levels of BCAAs. The approach from our pilot study can be expanded to test the metabolic implications of dietary BCAA manipulation.

Elevated fasting levels of branched chain amino acids (BCAAs: isoleucine, leucine, and valine) measured in venous blood have been associated with metabolic risk factors for type 2 diabetes (T2D), including obesity, insulin resistance, and elevated fasting glucose (1–5). Among individuals matched for age, sex, body mass index, and fasting glucose, levels of fasting BCAAs remain an independent risk factor for T2D even after controlling for measures of insulin resistance (6). Furthermore, for individuals with T2D, reductions in insulin resistance following gastric bypass surgery mirror reductions in fasting BCAA levels (7). These findings suggest that fasting levels of BCAAs are related to altered glycemic metabolism in T2D. However, experimental approaches are needed to clarify whether changes in fasting levels of BCAA directly affect or are only markers of metabolism in humans.

In animals, dietary manipulation of BCAAs can alter glycemic metabolism. In obese mice, nutritional interventions that increase all three BCAAs in the diet led to increased fasting levels of all three BCAAs and promoted insulin resistance (3). A second study that supplemented the diet of mice with only leucine observed increased fasting levels of leucine but decreased fasting levels of isoleucine and valine and decreased insulin resistance (8). These studies demonstrate that, in animals, dietary manipulation of BCAA intake can directly influence fasting levels of BCAAs and that the effect on metabolism may be dependent upon the manipulation of one or all BCAAs.

No human study to date has examined the effect of dietary manipulation of BCAA intake on fasting levels of BCAAs. One group has demonstrated that intravenous infusion of amino acids (containing BCAAs and non-BCAAs) acutely worsened insulin sensitivity (9). Yet, this study leaves unanswered whether BCAAs or other amino acids were responsible for changes in insulin sensitivity and whether a more physiological manipulation of BCAAs (i.e. through diet) would influence circulating fasting levels of BCAAs. If dietary manipulation of BCAAs influences fasting BCAA levels, then this approach could be applied in future studies as a research tool to explore the mechanisms linking fasting levels of BCAAs to altered metabolism or as novel methods for T2D prevention or treatment. We hypothesized that a structured nutritional intervention, using differences in BCAA intake, would alter fasting BCAA levels in healthy volunteers independent of other factors.

## Methods

### Study participants

**Fig. 1:**
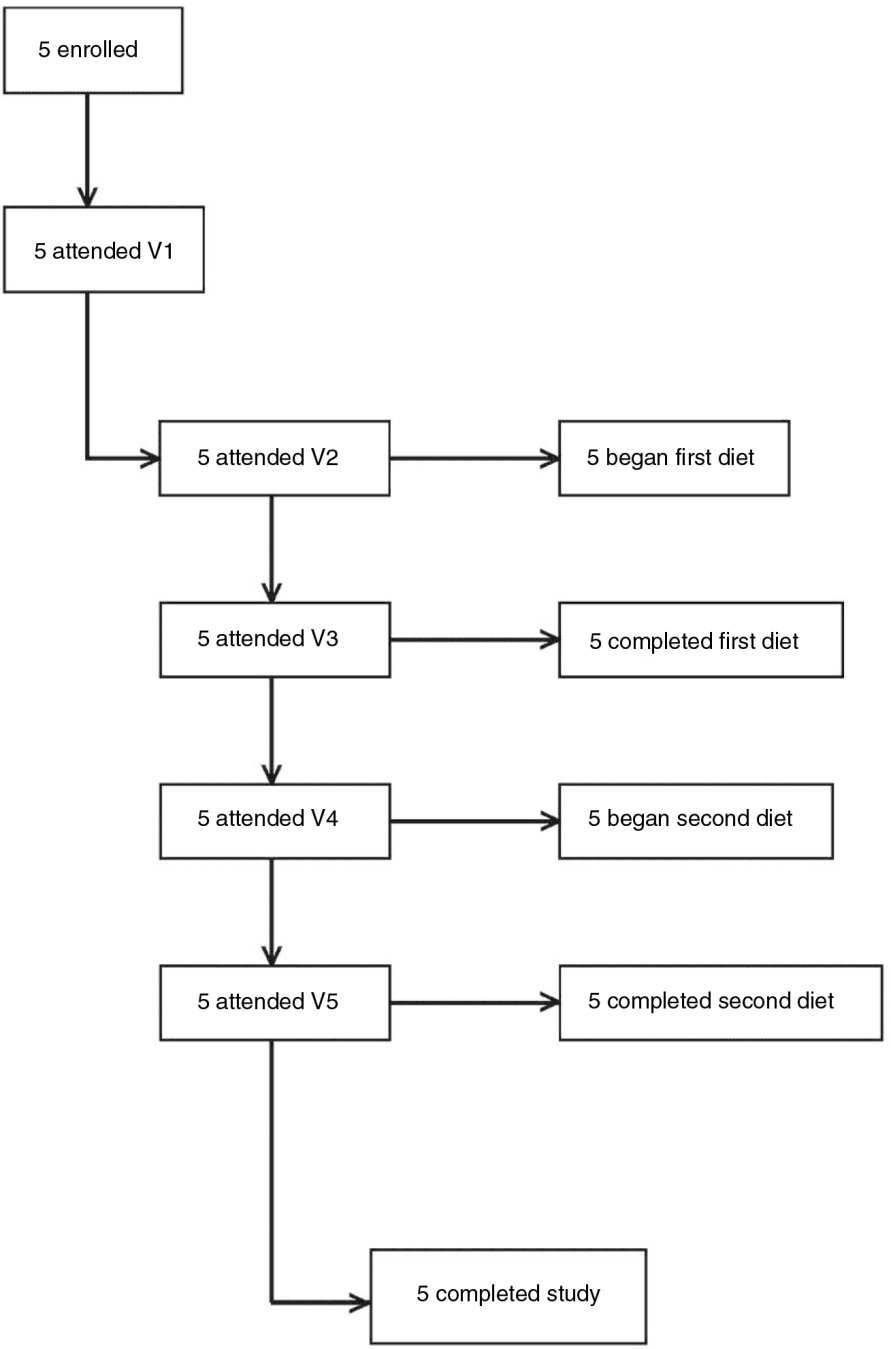
Study enrollment and completion.

We recruited and enrolled five healthy, adult men, age 20–40 years, into this single-center, single-blind, crossover nutritional intervention study. This cohort size was selected to provide 75% power to detect a difference in fasting BCAAs at alpha=0.05, assuming an effect of diet as large or larger than that observed in prior animal studies (3). Enrollment and completion is summarized in Fig. 1. Exclusion criteria included chronic medical conditions, such as T2D or hypertension, medication, herbal, or vitamin supplement during the month prior to enrollment, body mass index less than 20 kg/m^2^, change of weight by more than five pounds during the month prior to enrollment, use of nicotine-containing products during the study, use of drugs of abuse, conditions causing intestinal malabsorption, and inability to adhere to a prespecified meal plan, including standardized meals, abstinence from alcohol, and limitation to one caffeinated beverage per day. An initial exclusion threshold of BMI greater than 25 kg/m^2^ was changed to 28 kg/m^2^ to facilitate recruitment.

The protocol was approved by the local institutional review board, and informed consent was obtained from all study participants. The trial was registered on Clinical Trials.gov (NCT02110602) prior to enrollment. Participants were recruited through local advertising between March and July 2014, and all were compensated for their study involvement.

### Study protocol and laboratory measurements

Participants attended five visits at the Massachusetts General Hospital Clinical Research Center (CRC); participants arrived to each study visit after having fasted for at least 8 h. At Visit 1, weight and fasting blood glucose were measured from a venous sample and a full medical history was obtained. A research nutritionist interviewed each participant, calculated daily caloric requirements using the Mifflin St. Joer equation, and cross-checked the output with 25–30 kilocalories per kilogram of participant body weight. Activity factors were based on the results of the Paffenbarger questionnaire. This information was used to construct the study diets with adequate calories personalized to each participant. Between Visits 1 and 2 (an approximately 2-week period), participants consumed their native diet and completed a 4-day food log, which was used to estimate nutrient content of the native diet. Food logs were not used to estimate caloric requirements for the study diets.

At Visit 2, the 4-day food log was reviewed with assistance from a research nutritionist. Weight and fasting blood work for measurement of venous glucose, insulin, and BCAAs was performed just prior to starting the first study diet. Glucose was measured with a hexokinase assay (Roche, Indianapolis, IN); insulin was measured using a radio-immunoassay (Beckman Coulter, Fullerton, CA); and BCAAs were measured using an established platform (see *Measurement of BCAAs* below). After these measurements were completed, participants were provided with 4 days of meals and supplements for the first study period.

Breakfast for the first study period was eaten under observation in the CRC during Visit 2, and the remainder of the diet was consumed outside of the CRC. Visit 3 occurred at the CRC 3 days after Visit 2. At Visit 3, weight and fasting blood work for venous glucose, insulin, and BCAAs was repeated to assess values after completing the first study period. Any unfinished food or supplements were returned, and each participant completed an anonymous survey on aspects of adherence and quality of the first study period.

The participant then began a 3-day ‘wash-out’ of the first study period, during which he ate his native diet. The participant returned to the CRC for Visits 4 and 5, at which the activities from Visits 2 and 3, respectively, were repeated for the second study period.

### Low and high BCAA content dietary interventions

All participants received both the low and high BCAA content diets. Diets were provided in random order, and participants were not told in what order the interventions were provided. The elements of each dietary intervention are shown in Table 1. Foods and supplements were provided in take-out containers that could be stored, refrigerated and heated, if needed, by the participants.

**Table 1:** Elements of the controlled study diets

	Low BCAA Diet		High BCAA Diet	
		
	Day 1, 3	Day 2, 4	Day 1, 3	Day 2, 4
Breakfast	Coffee or tea with cream and sugar	Coffee or tea with cream and sugar	Coffee or tea with cream and sugar	Coffee or tea with cream and sugar
	Blueberry muffin	Waffles with syrup and butter	Blueberry muffin	Waffles with syrup and butter
	Nutrigrain bar	Applesauce	Nutrigrain bar	Applesauce
	Mandarin orange sections		Mandarin orange sections	
Lunch	Portobello, eggplant and pepper sandwich	Vegan lasagna	Portobello, eggplant and pepper sandwich	Meat lasagna
	Canned peaches	Tossed salad with oil and vinegar	Canned peaches	Tossed salad with oil and vinegar
	Mixed vegetables	Fresh orange	Mixed vegetables	Fresh orange
	Graham crackers		Graham crackers	
Dinner	Portobello burger	Stuffed pepper	Portobello burger with cheese	Stuffed pepper with cheese and kidney beans
	Potato chips	Mixed vegetables	Potato chips	Mixed vegetables
	Hard candy	Hershey kiss	Hard candy	Hershey kiss
	Fresh apple		Fresh apple	
Supplement	MSUD Express	MSUD Express	AjiPure BCAA	AjiPure BCAA

The dietary components for each day of the low and high BCAA interventions are shown. Supplements were consumed at all meals. Meal plans were organized for each participant, and the amount of food and supplements were based on the estimated caloric requirement for each participant to maintain a stable weight.

The total calories of each meal plan were designed to meet the needs of each participant and assure weight maintenance during the two study diets. As the total caloric intake (and therefore total grams of protein) varied for each participant, the supplement levels in each nutritional arm were adjusted to achieve the target percentage of total protein from BCAAs at each meal.

BCAAs are found in most protein-containing foods; therefore, the food components of the study diet interventions were generally low in protein. To achieve a low BCAA content intervention with an amount of protein typical for an American diet, participants consumed maple syrup urine disease (MSUD) Express (Vitaflo; Alexandria, VA) with each prepared meal. This BCAA-free amino acid supplement allowed for the food to contain a low amount of protein from the BCAAs for the low BCAA content diet but to have equal calories from protein as compared with the high BCAA content diet. The low BCAA content diet was intended to provide approximately 1% of protein calories from valine and isoleucine and 2% of protein calories from leucine, mirroring the 1:2:1 ratio for valine:leucine:isoleucine observed in a typical American diet. To achieve a high BCAA content intervention with an amount of protein typical for an American diet, participants consumed L-isoleucine, L-leucine, and L-valine supplements (AjiPure BCAA, Ajinomoto; Fort Lee, NJ) with each prepared meal. The high BCAA content diet was intended to provide approximately 14% of protein calories from valine and isoleucine and 28% of protein calories from leucine, again mirroring the 1:2:1 ratio for valine:leucine:isoleucine observed in a typical American diet.

### Measurement of BCAAs

Fasting BCAAs were analyzed on a validated platform for amino acid measurement (6); the platform also allows for simultaneous measurements of all amino acids as well as additional sugars, nucleic acids, and serum metabolites. Plasma venous samples from all participants were analyzed together in one batch and in random order. Reference pooled plasma samples were analyzed with the study samples and were placed in the analysis queue at the initiation.

Liquid chromatography tandem mass spectrometer (LC-MS/MS) peak areas were integrated and relative quantitative information was used in these analyses. The signal-to-noise ratio (S/N) of the metabolite peak area was greater than 30 in every sample (average S/N=122, s.d.=51.8), and therefore exceeded thresholds typically used to define limits of detection (S/N>3) and quantitation (S/N>10). All LC-MS analyses were performed using a 4000 QTrap triple quadrupole mass spectrometer (AB SCIEX; Foster City, CA) coupled to either an 1100 or 1200 Series pump (Agilent Technologies; Santa Clara, CA) or an HTS PAL autosampler (Leap Technologies; Carrboro, NC) equipped with a column heater.

Internal standard peak areas were monitored for quality control, and individual samples with peak areas differing from the group by more than two standard deviations were re-analyzed. MultiQuant software (Version 1.1; AB SCIEX; Foster City, CA) was used for automated peak integration, and metabolite peaks were manually reviewed for quality of integration and compared against a known standard to confirm identity.

### Statistical analysis

Analyses were conducted using GraphPad Prism (version 5.00 for Windows; San Diego, CA).

The 4-day dietary intake data were analyzed using Nutrition Data System for Research software (version 2013, Nutrition Coordinating Center (NCC); University of Minnesota, Minneapolis, MN). Pronutra (version 3.4.0; Viocare; Princeton, NJ) was used to analyze the nutrient content of the study diets. All fasting BCAA measurements were log transformed to approximate a normal distribution. Mean and standard error were provided for all quantitative variables, and paired *t*-tests were used to compare the effects of study diets. A threshold of alpha<0.05 was the threshold for statistical significance.

## Results

Five participants were enrolled into the study and all completed both controlled diet interventions (Fig. 1). All volunteers were healthy men, with an average age (mean±SE) of 27.6±2.3 years and average BMI of 25.3±1.0 kg/m^2^. All participated in regular exercise at baseline and consumed at least two meals prior to starting the study.

Nutritional characteristics of the native (baseline) diet and study diets are shown in Table 2. The same information is displayed, on a per individual basis, in Table 3. Both the low and high BCAA content diet contained more calories (total), a greater percentage of calories from carbohydrate and a lower percentage of calories from protein as compared with the native diets of the participants. The low and the high BCAA study diets did not differ from each other in terms of total calories, or percent of calories from protein, fat, or carbohydrate.

**Table 2:** Nutritional composition of native diets and the low and high BCAA content interventions

	Native diet	Low BCAA diet	High BCAA diet
Total calories (kcal)	2000.2±341.5	2422.7±172.8	2494.1±183.5
% Carbohydrate	49.0±5.7	53.0±0.3	53.3±0.4
% Fat	31.1±4.7	30.8±0.6	31.1±0.7
% Protein	19.6±1.9	16.3±0.3	15.7±0.4
Valine (g)	5.1±1.0	1.5±0.1	14.0±0.6
Leucine (g)	7.7±1.5	2.1±0.2	27.1±1.0
Isoleucine (g)	4.5±0.9	1.2±0.1	13.8±0.5

The mean±standard errors for measures of the native diet and for the low and high BCAA content interventions are shown. Nutrient profile of the native diet was derived from a 4-day food log. The low and high BCAA interventions ware based on meal plans and food consumed.

**Table 3:** Baseline and intervention dietary characteristics for each study subject

	Participant S1			Participant S2			Participant S3			Participant S4			Participant S5		
					
	Native	Low	High	Native	Low	High	Native	Low	High	Native	Low	High	Native	Low	High
Total calories	1062	1781	1871	2168	2398	2405	2303	2549	2589	1452	2800	3000	3016	2586	2605
% Carbohydrate	60.1	53.9	54.6	41.1	52.1	52	39.9	52.7	52.8	65.6	52.6	53.3	38.2	53.5	53.7
% Fat	20.2	28.7	28.7	37.9	31.4	32	33	31.3	31.5	20.6	32.1	32.5	43.9	30.5	30.8
% Protein	19.7	17.4	16.8	20.9	16.6	16	25.5	16.1	15.7	13.8	15.3	14.2	18	16.1	15.6
Valine (g)	2.6	1.08	12	6	1.4	13.8	7.2	1.6	14.6	2.8	1.8	15.2	6.7	1.6	14.6
Leucine (g)	4.2	1.5	23.4	8.7	1.9	26.6	11.2	2.2	28.1	4.1	2.5	29.3	10.2	2.3	28.1
Isoleucine (g)	2.3	0.9	11.9	5.3	1.1	13.5	6.7	1.3	14.3	2.3	1.5	14.9	5.7	1.4	14.3
% Study diet consumed		89%	93%		100%	98%		98%	99%		100%	100%		98%	100%

The measures of the native diet and for the low and high BCAA interventions for are shown for each participant. Information from the native diet was based on self-report from a 4-day food record (with assisted review by research nutritionist) analyzed in 2013 data base version of Nutrition Data System for Research (NDSR). Information from the low and high BCAA interventions is based on the consumption of actual amounts of provided meals based on Pronutra version 3.4.0 analysis.

By design, the low BCAA and high BCAA diets differed from the native diet and from each other in BCAA content (Table 1). As compared with the native diet, the low BCAA diet had lower amounts of valine (*p=*0.006), leucine (*p=*0.005), and isoleucine (*p=*0.007), and the high BCAA diet had higher amounts of valine (*p<*0.0001), leucine (*p<*0.0001), and isoleucine (*p<*0.0001). Consequently, the amount of each BCAA was different in the low and high BCAA diet (*p<*0.0001 for all).

During the low BCAA diet, participants consumed 90% of the study meals and supplements and consumed less than one non-study meal. During the high BCAA diet, participants consumed 97% of the study meals and supplements and consumed less than one non-study meal. Participants adhered to not more than one coffee or tea per day and did not consume alcohol during both diets. Each participant maintained baseline levels of exercise during both study periods. Metabolic parameters remain unchanged during study diets. Fasting glucose and insulin at baseline were 89±3.02 mg/dL and 6.3±1.2 µIU/mL, respectively, and there was no change in either fasting glucose or insulin as a result of the low (*p=*0.52 and *p=*0.79, respectively) or high BCAA content intervention (*p=*0.51 and *p=*0.29, respectively). Weight at baseline was 81.4±5.2 kg and was slightly lower when compared with the weight following either the low BCAA intervention (80.7±5.2 kg, *p=*0.04) or high BCAA intervention (80.8±0.16, *p=*0.16); weight was not different at the end of the low and high BCAA content interventions (80.7±5.2 kg vs. 80.8±0.16 kg, *p=*0.54).

**Fig. 2:**
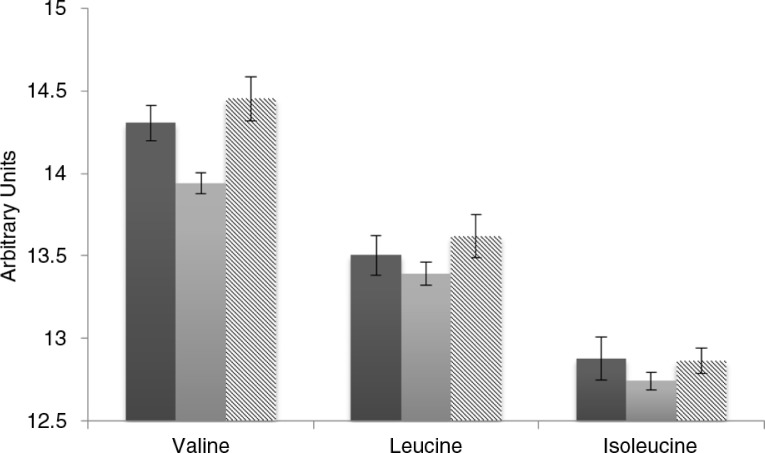
Fasting BCAAs levels following native diet and low and high BCAA content interventions. Shown are mean±standard error for fasting valine, leucine, and isoleucine on the native diet (dark gray bar) and following the low BCAA (light gray bar) and high BCAA (striped bar) interventions. Fasting BCAAs are presented in arbitrary units, representing area under the curve as measured on the mass spectrometer.

Figure 2 shows fasting BCAA levels on the native, low BCAA, and high BCAA diets across all participants. Following the low BCAA diet, there was a numerical decrease in fasting valine, leucine, and isoleucine from the native diet levels, but none of these comparisons were statistically significant. Following the high BCAA diet, fasting levels of valine, leucine, and isoleucine did not differ significantly from the native diet. The fasting levels at the end of the low BCAA vs. the high BCAA intervention were significantly lower for valine (*p=*0.02) and numerically lower for leucine (*p=*0.16) and isoleucine (*p=*0.38). Thus, a consistent pattern of modest differences in fasting BCAA levels was observed between the two study diets.

**Fig. 3:**
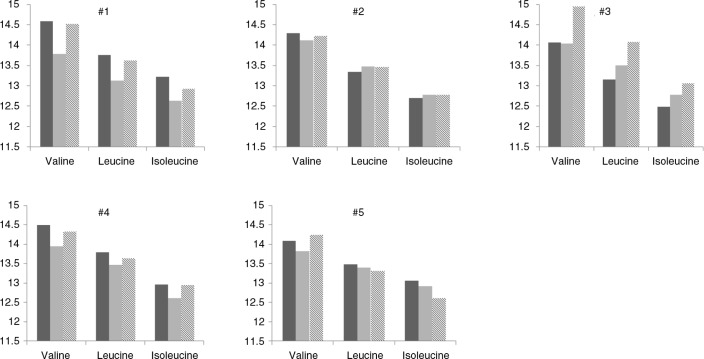
Fasting BCAAs levels for each participant following native diet and low and high BCAA content interventions. Shown are mean±standard error for fasting valine, leucine, and isoleucine on the native diet (dark gray bar) and following the low BCAA (light gray bar) and high BCAA (striped bar) interventions. Fasting BCAAs are presented in arbitrary units, representing area under the curve as measured on the mass spectrometer. Each number title corresponds to one of the five study participants.

The fasting BCAA levels for each participant, separately, are shown in Fig. 3. Following the low BCAA diet, the largest numerical decreases from baseline in fasting valine, leucine, and isoleucine were observed in Subjects 1 and 4; modest numerical decreases were observed in Subject 5; and minimal decreases or increases were observed in Subjects 2 and 3. Following the high BCAA diet, the largest numerical increases from baseline in fasting valine, leucine, and isoleucine were observed in Subject 3; minimal decreases or increases were observed in the other participants.

Subjects 1, 3, and 4 had the largest differences in fasting valine, leucine, and isoleucine following the low and high BCAA diets, and these differences followed the expected pattern of higher levels following the high BCAA diet as compared with the low BCAA diet. Subjects 2 and 5 had smaller differences in the amino acids following the low and high BCAA interventions, and these differences did not always follow the expected pattern.

There were no adverse events during the study interventions. The participants rated the quality of the dietary interventions relatively high (10-point scale; 0=worst, 10=best). For the low and high BCAA interventions, respectively, taste of food was rated (mean [lowest–highest]) 8.8 (6–10) and 9.1 (7–10), and taste of supplement was rated 5.5 (2.5–10) and 6.1 (1–10). Participants estimated that they could adhere to the low BCAA diet intervention for 5.25±0.8 weeks and to the high BCAA diet intervention for 6.75±1.2 weeks.

## Conclusions

In this pilot study, we tested whether a specific dietary intervention, using differences in BCAA intake, alters fasting BCAA levels independent of other factors. Participants tolerated the diets, rated them favorably on qualitative measures, and adhered closely to the prescribed interventions. Fasting valine was lower and fasting leucine and isoleucine trended lower after a low BCAA content diet as compared with after a high BCAA content diet. Group trends were for fasting valine, leucine, and isoleucine to be lower after the low BCAA diet and higher after the high BCAA diet as compared with levels on a native diet, but these effects were not consistent across subjects.

While the nutritional intervention in this study had modest effect, our findings suggest that interindividual differences, not characterized in this study, may have a more important role than dietary intake in determining fasting levels of BCAAs. This result is consistent with larger observational studies that have found no association between BCAA intake, as assessed by dietary recall, and fasting BCAA levels (6). Genome-wide association studies have found that genetic factors are responsible for determining up to 20% of BCAA levels; clinical factors, including age, sex, blood pressure, body mass index, and the presence of specific medical conditions, are responsible for an additional 30% of isoleucine and leucine levels and an additional 20% of valine levels (10). Thus, despite being essential amino acids, no more than 50% of the levels of isoleucine or leucine and no more than 60% of the levels of valine are influenced by diet alone.

Fasting levels of BCAAs are strongly influenced by protein degradation (11) and uptake of BCAAs by muscle (12–18). In the post-absorptive state, BCAAs are released from muscle and contribute to the fasting levels (19, 20). Exercise influences BCAA turn-over in muscle (21), which can be affected by amino acid intake (22, 23). Thus, any effort to maximally change fasting BCAAs through diet would likely also need to limit physical activity and other contributors to muscle utilization and breakdown. The absence of restriction on physical activity in this study may also explain why the effects of a controlled diet on circulating BCAAs were modest.

These results indicate that application of only a low or high BCAA content nutritional intervention in the short term without physical activity restriction is unlikely to result in a consistent decrease or increase, respectively, in circulating BCAAs from a native diet across individuals. Furthermore, because participants indicated they could adhere to either diet for less than 8 weeks, this approach is unlikely to be effective for prevention or treatment of chronic metabolic conditions.

However, our pilot study showed that modest differences in fasting BCAAs can be achieved in groups of individuals by comparing levels after a low BCAA diet and a high BCAA diet. The randomized, crossover design of this study increases the likelihood that any observed group differences were related to the intervention itself. While a statistical separation in fasting valine levels on a low versus high BCAA diet was achieved in this study with five individuals, larger studies using our interventions could achieve this separation for fasting leucine and isoleucine as well. Assuming a similar effect size as observed in this study, we would have 85% power to detect a significant difference in fasting leucine and isoleucine levels with 16 or 47 subjects, respectively. A study of such size could use the nutritional intervention from this study to explore mechanisms linking fasting BCAAs to altered metabolism.

The nutritional intervention platform developed here may be applied to other amino acids and nutrient metabolites. Our approach is flexible, incorporates food and specific supplements, and, because we used a metabolite profiling platform that can quantify amino acid and non-amino acid metabolites simultaneously, permits measurement of a broad range of dietary hypotheses. Whether fasting levels of other amino acids or nutrient metabolites with possible metabolic effects can be more easily modulated through dietary intake should be tested in different experiments. Thus, our platform offers a structured method of testing the influence of controlled dietary manipulation on fasting levels of specific nutrient metabolites and their implication on different conditions.

Strengths of this study include a design of a novel controlled dietary intervention, crossover application in random order to the low and high BCAA diets so that each subject served as his own control, high adherence to study diet, and measurement of BCAA levels in the fasted state before and after each intervention. Still, we acknowledge limitations to our study. We studied healthy subjects and for only 4 days on each intervention. This pilot study provided data to demonstrate that our method could be applied to other populations. It is possible that dietary manipulation of BCAA would be more effective in obese or insulin resistant individuals, who are known to have dysregulation of BCAA metabolism (1, 3), or would be more effectively in modulating BCAAs over long periods of a controlled diet. Second, we measured BCAAs only in the fasted state and only from venous blood. It is possible that, while on low or high BCAA content diets, circulating levels are more divergent in the post-prandial state or in other biological compartments, such as muscle. We used fasting values from venous samples as they are the ones previously identified as metabolic risk factors in obesity (1, 3), pre-diabetic states (4), and incident T2D (4, 6). Third, we did not restrict or control the amount of physical activity of the research participants. While participants were instructed to maintain baseline levels of physical activity throughout the study, differences in muscle turnover and metabolism may have influenced our results. While the goal of this study was to assess the influence of dietary intake alone on fasting BCAA levels, further studies using a dietary approach should also control physical activity to have maximal influence on fasting BCAA levels. Finally, all BCAAs were manipulated in the same diet. It is possible that a low or high isoleucine content diet, for example, would have been more effective at altering isoleucine levels than the one that manipulated all three BCAAs. Future studies could examine the feasibility of altering only one amino acid, particularly if it is of higher interest than others.

In conclusion, we constructed a novel nutritional intervention to test whether short-term intake of a controlled diet could alter circulating levels of BCAAs in this pilot study. The intervention was more effective at providing a modest difference in BCAA levels following the low and high BCAA diets than in consistently lowering or raising circulating BCAAs from the native diet. Future studies can utilize the interventional construct developed here, along with physical activity restriction, to test the effect of BCAA or other amino acid manipulation under different experimental settings.
